# A Health Equity Approach for Implementation of JYNNEOS Vaccination at Large, Community-Based LGBTQIA+ Events — Georgia, August 27–September 5, 2022

**DOI:** 10.15585/mmwr.mm7143e4

**Published:** 2022-10-28

**Authors:** Alexander J. Millman, Damian J. Denson, Michelle L. Allen, John A. Malone, Demetre C. Daskalakis, Diane Durrence, R. Chris Rustin, Kathleen E. Toomey, Atlanta Black Gay Pride Festival Monkeypox Response Team, Atlanta Black Gay Pride Festival Monkeypox Response Team, Tracy Dabbs, Frederick Dobard-Gary, Paige E. Harton, Leah Hoffacker, Huriyyah Lewis, Sheila Lovett, Dewayne Crowder, A Vision, Brittany Johnson, Catherine Monroe, Linda O’Sullivan, Sandra Valenciano, David P. Holland, Joshua O’Neal, Audrey Arona, Dorian Freeman, Alana Sulka

**Affiliations:** ^1^Georgia Department of Public Health; ^2^Division of State and Local Readiness, Center for Preparedness and Response, CDC; ^3^CDC Monkeypox Emergency Response Team.; Georgia Department of Public Health; Georgia Department of Public Health; Georgia Department of Public Health; Georgia Department of Public Health; Georgia Department of Public Health; Georgia Department of Public Health; Hope; Cobb & Douglas Public Health; Cobb & Douglas Public Health; DeKalb County Board of Health; DeKalb County Board of Health; Fulton County Board of Health; Fulton County Board of Health; Gwinnett, Newton, and Rockdale County Health Departments; Gwinnett, Newton, and Rockdale County Health Departments; Gwinnett, Newton, and Rockdale County Health Departments.

Gay, bisexual, and other men who have sex with men (MSM) have been disproportionately affected during the 2022 U.S. monkeypox outbreak, with Black or African American (Black) MSM being the most affected demographic group (*1*). As of September 28, 2022, Georgia had reported 1,784 monkeypox cases; 98% of which occurred in males and 77% among Black persons (*2*). As of September 13, 2022, 60% of reported cases were among persons with HIV infection, and 50% of persons with monkeypox had a sexually transmitted infection within the past year (*3*). Because of racial disparities in the incidence of monkeypox cases and a large proportion of cases among MSM in Georgia, early vaccination beginning in July focused on improving equitable access by establishing new and leveraging existing partnerships with community-based organizations that serve affected populations, including persons with HIV infection. Despite these efforts, disparities persisted because of high demand and limited vaccine supply. The Georgia Department of Public Health (DPH) requested CDC support for a vaccine pilot and received an additional allocation of 5,500 doses of JYNNEOS vaccine for administration at events leading up to and throughout a Black gay Pride festival in Atlanta, a multiday event held Labor Day weekend (September 2–5, 2022). The event celebrates lesbian, gay, bisexual, transgender, queer or questioning, intersex, asexual, and other (LGBTQIA+) communities of color and hosts more than 125,000 attendees each year. Before the festival (as of August 24), 17,546 persons had been vaccinated in Georgia, of whom 96% were male, 34% aged 25–36 years, 44% Black, and 8% Hispanic or Latino (Hispanic) (*4*).

During August 27–September 5, Georgia DPH, in conjunction with metropolitan Atlanta local public health departments in five counties^†^ and local community-based organizations, administered the additional allocation of JYNNEOS vaccine. Vaccination events held before the festival (August 27–September 1) were located at local health department clinics and at venues acceptable and convenient for Black MSM, such as familiar large event spaces, bars, and clubs. During the festival (September 2–5), vaccine events were held during the day and after hours at health department clinics and at bars and clubs via mobile vans to increase broad reach and access. Georgia DPH staff members used social media, community-based organizations, and field outreach to promote vaccination events. Vaccines were administered by Georgia DPH, partner organizations, and local health departments. Patient demographic data were collected at the time of vaccination and entered into the Georgia Immunization Registry. Aggregate data were shared with CDC. This activity was reviewed by CDC and was conducted consistent with applicable federal law and CDC policy.^§^

During August 27–September 5, a total of 4,282 JYNNEOS vaccine doses (78% of the additional allocation) were administered. Two thirds (2,874) of doses were administered before the festival and one third (1,408) during the event. Overall, 2,886 (67%) doses were administered at 22 routine vaccination events at health department clinics, 702 (16%) doses at 20 mobile, community pop-up events, and 694 (16%) doses at one fixed location (a Georgia DPH-sponsored mass vaccination event). Among vaccine recipients, 93% were male, 55% were aged 30–49 years, 48% were Black, and 8% were Hispanic (Table). The proportion of Black persons receiving vaccine was higher during the festival (53%) than before the event (46%), but the proportion of Hispanic recipients was similar (7% versus 8%). Nearly one third (31%) of records were missing data on state of residence.

**TABLE:** Characteristics of recipients of JYNNEOS vaccine before and during a Black gay Pride festival in Atlanta (N = 4,282) — Georgia, August 27–September 5, 2022

Characteristic	No. (%)		
	Total Aug 27–Sep 5	Before festival Aug 27–Sep 1	During festival Sep 2–5
**Total**	**4,282**	2,874	1,408
**Self-reported sex**			
Female	**205 (4.8)**	153 (5.3)	52 (3.7)
Male	**3,970 (92.7)**	2,639 (91.8)	1,331 (94.5)
Other/Unknown	**107 (2.5)**	82 (2.9)	25 (1.8)
**Age group, yrs**			
0–17	**5 (<1)**	4 (<1)	1 (<1)
18–29	**614 (14.3)**	410 (14.3)	204 (14.5)
30–49	**2,353 (55.0)**	1,571 (54.7)	782 (55.5)
50–64	**920 (21.5)**	630 (21.9)	290 (20.6)
≥65	**390 (9.1)**	259 (9.0)	131 (9.3)
Ethnicity*			
Hispanic or Latino	**328 (7.7)**	224 (7.8)	104 (7.4)
Non-Hispanic or Latino	**3,872 (90.4)**	2,585 (89.9)	1,287 (91.4)
Unknown	**82 (1.9)**	65 (2.3)	17 (1.2)
Race*			
Asian	**132 (3.1)**	89 (3.1)	43 (3.1)
Black or African American	**2,069 (48.3)**	1,322 (46.0)	747 (53.1)
White	**1,625 (37.9)**	1,144 (39.8)	481 (34.2)
Other	**396 (9.2)**	276 (9.6)	120 (8.5)
Unknown	**60 (1.4)**	43 (1.5)	17 (1.2)
**State of residence**			
Georgia	**2,893 (67.6)**	1,996 (69.5)	897 (63.7)
Other	**52 (1.2)**	29 (1)	23 (1.6)
Unknown	**1,337 (31.2)**	849 (29.5)	488 (34.7)
**Dose**			
First	**2,516 (58.8)**	1,765 (61.4)	751 (53.3)
Second	**1,766 (41.2)**	1,109(38.6)	657 (46.7)

* Hispanic or Latino persons can be of any race. Other includes persons who identify as Native Hawaiian or other Pacific Islander, American Indian or Alaska Native, or multiracial, and persons who declined to report.

Vaccinating communities disproportionately affected by the monkeypox outbreak is important in stopping spread of *Monkeypox virus* and ending the outbreak (*5*). A community-based approach by a coalition of festival organizers, government entities, and LGBTQIA+ community advocates was successful at improving equitable monkeypox vaccination. This work highlights the value of health department and community-based organization in-person and virtual outreach to increase health equity. Challenges to an equitable approach to monkeypox vaccination included decreasing trends in vaccine demand by the time of the event (possibly attributable to historical vaccine hesitancy or stigma), ensuring second doses for out-of-state travelers, and recent concerns about receiving second doses because of skin discoloration associated with intradermal vaccine administration.^¶^ Georgia DPH is implementing additional events to help ensure that persons in Georgia receive their second JYNNEOS vaccine dose. As the vaccine supply increases, distribution strategies should continue to focus on eliminating disparities and removing barriers, especially for persons who might be hesitant to be vaccinated or uncomfortable being vaccinated at a large event.

## Footnotes

† Clayton, Cobb, DeKalb, Fulton, and Gwinnett counties.

§ 45 C.F.R. part 46, 21 C.F.R. part 56; 42 U.S.C. 241(d);5 U.S.C. Section 552a; 44 U.S.C. Section 3501 et seq.

¶ Updated clinical considerations for intradermal administration of JYNNEOS vaccine allow for more discrete anatomic locations such as the upper back below the scapula or at the deltoid.
